# On the Meaning of the Term “Symptom”

**Published:** 1855-10

**Authors:** C. A. Hunt

**Affiliations:** Urbana, Illinois


### Art. III.—ON THE MEANING OF THE TERM "SYMPTOM."

BY C. A. HUNT, M. D., OF URBANA, ILLINOIS.

The editor of the *Peninsular Journal of Medicine* cites us to Webster's definition of 'symptoms,' and afterwards gives his own, both of which he seems to consider unexceptionable. Webster says, "a symptom is a perceptible change in the body or its functions which indicate disease." The editor says, "every intelligent physician knows that a symptom means a deviation from the normal condition of an organ or function, perceptible to the patient and to others, which indicates the presence of disease, and assists the judgement in forming an opinion of its seat, nature, etc, etc." I ask the privilege of differing on this point with both of these authorities, for I consider them essentially wrong in the whole.

"*A symptom, a perceptible change in the body or function.*" Suppose we discover in a certain part of the system an abscess, a tubercle, or a caries; would we not at once confess it to be a perceptible change in that part of the body as well as the functions of the part; yet who would contend that it was a symptom and not a disease itself? If we are called to a case of what is termed "idiopathic fever," this fever is the changed condition of parts and of functions by which it becomes entitled to the appellation of disease. This perceptible change in the part and function is all we can recognize in a disease, for when the change ceases to be made in the body or its constituted parts, the functions of this body, it must then have reached the elements of the body and constitutes decay. But we see in the editor's definition precisely the same thing although it is more prolix and not so clear. The symptom according to him is "a deviation from the normal condition of an organ or function." Now the organ means the material structure, and the function, the office that it performs, and the deviation from the normal condition of the substance or the function is in all cases the pathological condition, or the disease in question. These three pathological states may exist, either fever, congestion or inflammation. This hyperemic condition incident to each is termed a disease, for they are express pathological conditions and are the perceptible changes of the organ or its functions, or of the body and its functions, yet who would call that condition a symptom?

Symptoms are the only evidence that we can possess of internal disease, by which alone we are enabled to arrive at the pathological condition. The first impress made by changing function or structure is upon some one or more parts of the economy, and must reach the brain by the afferent nerves, in the same manner that impressions from external nature do, to wit: by the sensory ganglia. These impressions in either case are resolved into sensations, the one normal, the other abnormal—the one received by organs endowed with nerves especially designed for that object, the other received by nerves whose office is the consequence of normal impressions from the interior economy and which become deranged by the infringement of foreign matter, hence any injurious impression is conveyed and received as such, and disbursed as such, and the sensation evolved is not normal function, but deranged feeling and function, and on the one hand is aggregated into pain by unequal distribution, and on the other is depraved into atrophy, etc., by the other excess. The manifestations of these impressions, made and disbursed are secondary to the cause in action which produced them, and become the only source of our knowledge of this internal change,—and are the symptoms proper; and in every case the manifestation comes secondary to the changed and changing condition of the part and is a reflection. We can get no symptom of deranged action except the reflex action; for impressions are not made from the brain and spinal cord to the diseased part, but from the part affected to the nervous center; hence change of matter and phenomena must precede the evidences to the bystander as well as the patient. Even in case of an external wound, where, for instance, the skin is pinched, the contortions of the face and other motions are symptoms of pain, and the pain is a symptom of abnormal phenomena —but the part compressed is where the "perceptible change" is made in the body and its functions, and that is the spot which is diseased. This is precisely the *modus agendi* from the interior of the organism. Every abnormal impression proceeds from derangement made upon the matter or the force which acts through it, and is represented to the brain in the same unfriendly light and is reflected therefrom emerging into symptoms varying as the general tonicity is presented or not, and as the organs and functions vary in texture and office. In perfect health there is perfect satisfaction and no new phenomena are presented to the senses, but on the contrary unmixed harmony prevails, which, like a galvanic circle, is not disturbed, and gives no evidences of its latent force until obstructed by some foreign cause. When the disturbance begins the manifestation given through a responsive action is the first evidence we can have of the foreign obtrusion and this is the symptom. What then is the definition of a symptom? I answer, *the manifestation of abnormal phenomena.**

---

*A symptom is any change perceptible to the senses, occurring in any organ or function, and connected with the exintence of disease.—*Chomel.—D. W. Y.*

It is important for us to understand that the symptoms or signs of disease are never to be taken in the like sense with that in which the signs of external things are often regarded. The buoy which, floating in the river, marks its navigable tract; the bell which, by striking, denotes the lapse of time; the stone by the wayside, which tells us how far we have come, and how far we have yet to go; these are most important signs, and of indispensable service to us all, but they have no natural connexion with the things they are made to indicate. They are mere expedients of conventional meaning and use. Navigable rivers, and time and space, would still exist, though there were neither buoy, nor bell, nor milestone, in the universe.

There is nothing that we call the symptoms of a disease, which does not oontain within itself much more than a mere sign. Heat, pain, redness, swelling, are called

In carrying out the illustration of symptomatology I will define also what I mean by disease of the abnormal phenomena of which I hold the symptoms to be the manifestation. A disease is the evidence of deranged matter or deranged force which acts through it. In a word the deranged matter or force, or both, develope the new phenomena, known as disease and the manifestation is the symptom. Are symptoms then, the only expression of disease? I answer they are—in saying which I mean internal disease, for those on the exterior can be known by the direct phenomena as presented to the sight without any accompanying symptoms. The symptoms of the disease then are the representatives of deranged action or phenomena, as words are representatives of thought or motives. The expressions of each are developed through mechanical force under increased or diminished muscular action; language through the voluntary muscles, and symptoms through both voluntary and involuntary, in proportion to the part affected. The muscles and all contractile fibres including the heart, capillaries, absorbents, excretory ducts, etc., become the media of symptomatic expression, while the muscles of the tongue, face, fauces, larynx, etc., are those used in language. Words are used to convey and are the evidences of impressions, normal or abnormal, those from the interior economy and those from external nature, while symptoms are evidences only and designed to convey abnormal impressions, having arisen out of disease or derangement.

---

the signs of inflammation; but nature does not intend by them barely to intimate that inflammation exists; they are essentially connected with the processes she is carrying on.

Thus at early dawn we point to the first glimmering in the east, and call it a sign of the rising sun; but it is more—it is an emanation from his beams. We look at the cloud above our heads, and say it is a sign of rain; but it is the gathering of the waters themselves.

Concerning symptoms I would nevertheless remark (what is very important to be borne in mind), that they stand in different relations to the disease to which they belong. They may flow out of the disease, so as, in idea at least, to be separable from it; or they may be involved in the disease, so as to be identical with it. The difficult respiration, the cough, the sputa, the emaciation, the hectic, are the symptoms of phthisis, and are distinguishable from the disease itself. They are the signs of something beyond themselves, which we do not see—viz., tubercles of the lungs. But the symptoms which denote an intermittent fever are the same which constitute

A symptom then being the representative of the abnormal phenomena must always arise after the inception of the phenomena and end with their cessation. If disease has created, or is represented by a symptom, the evidence of its existence must cease with the cessation of the representative. When a disease has ceased to be represented, or ceased to develope a symptom, it must then be as harmless to the organism as before it developed one. But inasmuch as we have no other guide to the disease except through the symptoms, we must content ourselves with believing disease to have ended when it shows no more evidence of its existence either to us or to the patient. We, therefore, could not be justified in treating an organism for any disease when all the symptoms have ceased, for the exhibition of a remedy (if it have any effect at all) must at such a time, if not before, create symptoms of its own which in turn must be overcome by another remedy, or by the recuperative energies of life. Parts of the economy may at one time by excessive excitement eclipse symptoms in another part, yet when the violence of these shall have abated, those from the other part may become apparent, and in turn obscure the first by excess of action, yet the eclipse is no evidence of the cessation of the symptoms in either, but the total cessation of symptoms from each or both would be coincident with the disappearance of the new phenomena, to wit: the disease itself.

The commonly recieved views that disease (say fever,) holds an independent sovereignty in the economy, and gains its admission

---

the disease. We have no idea of an intermittent apart from the rigor, the heat, and the perspiration. The same may be said of other fevers, and almost all diseases not organic, in which, if you seek to separate the symptoms from the disease, you must resort to theory for the purpose, and conceive an action of a certain kind prior to the actions which constitute the symptoms, and are productive of them.

There are then symptoms which, in the plain and intelligible sense, are signs and tokens of the disease that exists separately and distinctly from them; and there are symptoms which, however they may be spoken of as signs merely, are, nevertheless all that we know of the disease itself. The disease is the symptoms; and the symptoms are the disease.

True: but it can hardly be conceived that they are in reality the same. Yet it is better that they should be so regarded, than that we should go beyond our knowledge in attempting to distinguish them. Better to see in fevers only a certain combination of symptoms, than to run wild about a debility of the nerves, a spasm of the extreme vessels, or a peccant matter in the blood.

as an independent agent is very nearly deserving the epithet of superstition. On the contrary when a disease exists it is a phenomenon flowing constantly from deranged matter or deranged force, just as health or harmonious function is the invariable result of the normal condition of the matter and the force which developes life out of it. Life and health are but phenomena flowing from force acting through matter of a certain arrangement and certain integrity, and when that integrity and arrangement are violated, derangements aggregating into disease as certainly result, as health eventuates under the former circumstances, because in the economy the vital force propels perpetually phenomena which must be identified as normal or abnormal in the proportion that the relations of force to matter or matter to force are preserved or deranged. If my position is comprehended by the reader, he must see that disease is a phenomenon differing from the legitimate phenomena of normal force or matter called health, and that these phenomena are converted from one to the other by the derangement of the force or the matter through which the force acts. Now it follows that if health is known by sensation, by emotion, by feelings, and by impressions, the opposite condition, disease, is also known through the same media and the feature, the expression, the representation, which identifies this deranged phenomenon are the symptons, to-wit: The manifestation of deranged phenomena. When the indications of health fail to be presented the phenomena are termed disease. When the indications of organic life thus fail it is termed death, yet the force or the matter

---

Again: I would remark that it is often most difficult to draw the line between what is disease, and what is symptom; and that the same conditions may in different cases become now one and now the other. A dropsy, or a hæmorrhage, are sometimes primarily and essentially the disease. Sometimes they are secondary, and incidental to the real disease, and are themselves only symptoms.

How impossible, then, must it be to give any definition of a symptom which shall be philosophically true, and at the same time satisfy every sense in which it is practically regarded!

From the imperfection of our knowledge the whole subject of semeiology is beset with philosophical difficulties; and no advantage will be gained from conducting our inquiry concerning it in a stricter method that its own nature will bear.

Whatever it is that bespeaks the presence of diseases, or denotes their nature or their seat, or moreover, whatever indicates the proper method of treating them, may be equally regarded in the character of a symptom.—*Latham.*—*D. W. Y.*

are not lost but only changed in relation. From the preservation of the force and matter in their integrity flows health; from their derangement flows disease; from their destruction flows death, and the expressions, and features, and representations of each termed symptoms, are marked and must exist with the existence of each and end with the termination of each.

*Urbana, Ill. Oct.*, 1855.

